# Treating intrauterine adhesion using conditionally reprogrammed physiological endometrial epithelial cells

**DOI:** 10.1186/s13287-022-02860-w

**Published:** 2022-05-03

**Authors:** Siyu Xia, Ming Wu, Xinhao Zhou, Xiu Zhang, Lina Ye, Kang Zhang, Yiyi Kang, Jun Liu, Yunci Zhang, Wang Wu, Dirong Dong, Hong Chen, Hui Li

**Affiliations:** 1 School of Basic Medical Sciences, Wuhan University, Wuhan, 430071 Hubei China; 2 Wuhan University Shenzhen Institute, Shenzhen, 518057 Guangdong China; 3 Department of Obstetrics and Gynecology, Zhongnan Hospital of Wuhan University, Wuhan, 430071 Hubei China

**Keywords:** Mouse endometrial epithelial cells (MEECs), Conditional reprogramming (CR), Intrauterine adhesion (IUA), Regeneration and repair, Estrogen/progesterone receptor (ERα/ PR)

## Abstract

**Background:**

There is unmet need for effective therapies of intrauterine adhesions (IUAs) that are common cause of menstrual disturbance and infertility, since current clinical procedures do not improve prognosis for patients with moderate to severe IUA, with a recurrence rate of 23–50%. Stem cell-based therapy has emerged as a therapeutic option with unsolved issues for IUA patients in the past few years. Primary endometrial epithelial cells for cell therapy are largely hampered with the extremely limited proliferation capacity of uterine epithelial cells. This study was to evaluate whether IUA is curable with conditionally reprogrammed (CR) endometrial epithelial cells.

**Methods:**

Mouse endometrial epithelial cells (MEECs) were isolated from C57BL female mice, and long-term cultures of MEECs were established and maintained with conditional reprogramming (CR) method. DNA damage response analysis, soft agar assay, and matrigel 3D culture were carried out to determine the normal biological characteristics of CR-MEECs. The tissue-specific differentiation potential of MEECs was analyzed with air–liquid interface (ALI) 3D culture, hematoxylin and eosin (H&E) staining, Masson’s trichrome and DAB staining, immunofluorescence assay. IUA mice were constructed and transplanted with CR-MEECs. Repair and mechanisms of MEECs transplantation in IUA mice were measured with qRT-PCR, Masson’s trichrome, and DAB staining.

**Results:**

We first successfully established long-term cultures of MEECs using CR approach. CR-MEECs maintained a rapid and stable proliferation in this co-culture system. Our data confirmed that CR-MEECs retained normal biological characteristics and endometrium tissue-specific differentiation potential. CR-MEECs also expressed estrogen and progesterone receptors and maintained the exquisite sensitivity to sex hormones in vitro. Most importantly, allogeneic transplantation of CR-MEECs successfully repaired the injured endometrium and significantly improved the pregnancy rate of IUA mice.

**Conclusions:**

Conditionally reprogrammed physiological endometrial epithelial cells provide a novel strategy in IUA clinics in a personalized or generalized manner and also serve as a physiological model to explore biology of endometrial epithelial cells and mechanisms of IUA.

## Introduction

The endometrium comprises of columnar epithelium and a substantial vascularized stroma. The columnar luminal epithelium extends to form a pseudo-stratified epithelium which lines the endometrial glands [1]. During menses, the epithelial cells re-epithelialize the exposed surface and regenerate the new functionalis under the control of estrogen, while the glands remain in the basalis layer [2]. The normal endometrium structure is essential for embryo implantation and pregnancy maintenance. Up to 20–25% of patients occur intrauterine adhesion (IUA) or fibrosis, also known as Asherman’s Syndrome, after pregnancy-related curettage or hysteroscopic myomectomy [3, 4]. Mechanical trauma or infection to the basal layer of endometrium often results in partial or complete fibrosis and obliteration [5]. IUA often leads to hypomenorrhoea or amenorrhoea, menopause, pelvic pain, infertility or recurrent pregnancy loss [3]. Currently, hysteroscopic adhesiolysis is the commonly used treatment for IUA patients [6]. In order to prevent recurrent adhesion, treatments such as placing an intrauterine device (IUD)/Foley’s catheter balloon/hyaluronic acid or estrogen/progesterone are followed after adhesiolysis [7]. However, these procedures lack evidence from large-scale randomized clinical trials and pregnancy follow-ups [8]. These various therapies exhibit poor efficacy, systemic side effects, and high recurrence rate due to the intricate mechanism of basal layer production and loss of most endometrial cells in the severe damage cases [4, 6, 9]. The efficient complementary therapies after adhesiolysis are urgently needed.

The initiation step of repair is re-epithelialization of the endometrial surface, which is complete within 48 h after the beginning of menstruation and precedes stromal expansion [10]. Rapid epithelial repair is critical for prevention of fibrosis and scar formation [11]. The specific population of endometrial epithelial cells with stemness are capable of regenerating endometrial epithelial structures [12]. Cell-based therapy has emerged as a therapeutic option for patients with Asherman’s syndrome in the past few years. For example, transplanted cells may provide morphological and functional benefits including trophic support, cell replacement, regeneration of endogenous cells, interactions with endogenous cells, immunosuppression/anti-inflammation [13, 14]. Stem cells derived from bone marrow, mesenchyma, menstrual blood, placenta, and cord blood have been used as in the clinical and preclinical animal models for uterine repair and regeneration [3, 7, 9, 15]. However, there are several unsolved issues in applications of stem cells, including precise induction of differentiation, genomic instability, tumorigenesis, ethic problems, and unclear molecular mechanisms [16–20].

Although the existence of uterine epithelial cells with stemness was hypothesized decades ago, the first functional report for putative endometrial epithelial or stromal stem cells was in 2004 [21]. Recently, the specific markers have been identified for epithelial stem cells in human (EpCAM, E-cadherin, Mucin1) and murine (EpCAM, CD44) endometrium [12, 22, 23]. However, utilization of endometrial epithelial cells for cell therapy is hampered due to the extremely low yield and limited proliferation capacity of uterine endometrial cells [11]. Conditional reprogramming (CR) technique allows to establish primary and long-term continued cultures of normal epithelial cells derived from small sizes of tissue samples (as few as four viable cells) by using irradiated murine fibroblast feeder cells and a Rho kinase inhibitor (Y-27632) [24, 25]. Different from traditional immortalized cells, CR cultured cells are established without genetic manipulation, such as transduction of exogenous viral or cellular genes. Therefore, CR cultured cells are genomic stable and non-tumorigenic [24]. CR cells also retain lineage commitment and the ability to differentiate into the tissue of origin when withdrawn CR culture condition [24, 25]. CR cells maintain a rapid and stable proliferation in co-culture system and easily meet the requirement of efficacy demanded by clinical transplantation [26]. These advantages have shown great potential for cell-based therapy [26–28]. To our knowledge, there is no report regarding application of CR cells in repair of IUA.

In the present study, we established long-term culture of mouse endometrial epithelial cells (MEECs) by CR method. MEECs retained normal biological characteristics and tissue-specific differentiation potential. Moreover, MEECs expressed estrogen and progesterone receptors and possessed response to hormones in vitro. Transplantation of MEECs successfully repaired the injured uterine endometrium and significantly improved the pregnancy rate of IUA mice.

## Materials and methods

### Cell isolation and propagation

**Fig. 1:**
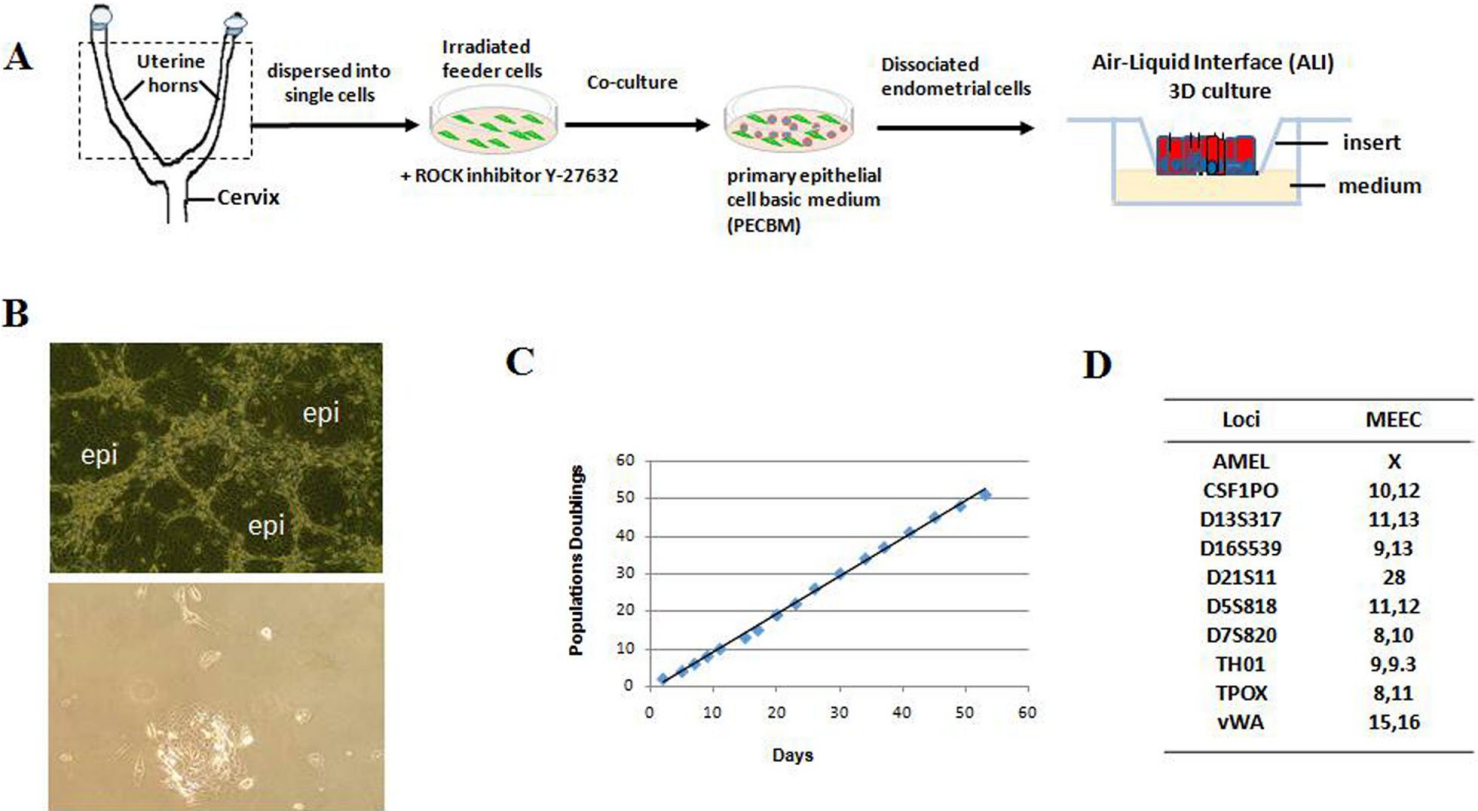
Continued cultures and expansion of mouse endometrial epithelial cells (MEECs). **A** A schematic diagram to illustrate the isolation, expansion, and ALI 3D cultures of MEECs. **B** The morphology of MEECs in CR condition (upper panel) and regular condition (lower panel). The fresh murine uterus tissues were minced into pieces and dispersed into single cells. And then cells were plated in primary epithelial culture basic medium (PECBM) and co-cultured with irradiated mouse fibroblasts as described in “Materials and methods” section or DMEM supplemented with 10% FBS. The morphology of MEECs was photographed under the phase contrast microscope. Magnification 10 ×. MEECs were marked as “epi.” **C** The growth curve of MEECs. The cell growth curve was plotted as accumulated population doublings versus time (days). **D** Cell identification (Cell ID)—the short tandem repeat (STR) analysis of the MEECs. STR analysis showed that 10 STR loci of MEECs do not match any other cell lines registered in the database

The uterine horns were collected from one 8-week-old C57BL female mice (Wuhan University Center for Animal Experiment). The Animal Ethics Committee of Wuhan University Center for Animal Experiment approved this study. Cell isolation and culture procedures were carried out according to the previous studies with minor modifications [24, 25, 29, 30]. In brief, fresh murine uterus from one female mouse was dissected and the uterine horns were collected as illustrated in Fig. 1A. Uterine horns were minced into pieces and dispersed into single cells by digestion with collagenase (StemCell, Vancouver, BC, Canada) plus trypsin. The primary mouse endometrial epithelial cells were co-cultured with irradiated mouse fibroblast 3T3 cells (J2 strain) (YongTech, Shenzhen, China) in primary epithelial culture basic medium (PECBM). PECBM contains DMEM and nutrient F-12 Ham (3:1) (v/v) (Sigma-Aldrich), supplemented with 5% FBS (GIBCO), 2 nM triiodothyronine (Sigma), 0.5% insulin–transferrin–selenium reagent (Life Technologies), 5 μg/ml transferrin (Life Technologies), 10 ng/mL epidermal growth factor (Sigma), 0.4 μg/mL hydrocortisone (Sigma), 1 nM cholera toxin (List Biological Labs), 0.5 μg/mL amphotericin B (Fungizone; Bristol-Myers Squibb), 40 μg/mL gentamicin (Gentacin; Life Technologies), and 5 to 10 mol/L Y-27632 (Enzo Life Sciences). The cells were cultured at 37 °C in a humidified incubator, with 5% CO_2_. The passaging CR cells was trypsinized in two steps [31]. The initial trypsinization was for removing feeders, and another trypsinization was for detaching epithelial cells. The cell growth curve was plotted as accumulated population doublings versus time (days) [29].

### Short tandem repeat (STR) analysis

Cellular genome DNA of MEECs was extracted with a commercial kit (Tiangen, China). Short tandem repeat (STR) analysis (DNA fingerprinting) was performed commercially as described previously [30, 32]. Co-amplification and three-color detection of 10 loci (9 STR loci and the X-chromosome-specific loci) were recognized.

### DNA damage response analysis

Cells were treated with or without 0.5 nM actinomycin D (Act D) for 24 h. SDS polyacrylamide gels electrophoresis was conducted and then transferred electrophoretically on to a 0.2 µm polyvinylidenedifluoride (PVDF) membranes (Immobilon-NC, Millipore), specifically probed with following primary antibodies: mouse anti-p53 (1:1000, Santa Cruz Biotechnology, CA, USA, sc-126), mouse anti-p21 (1:200, Santa Cruz Biotechnology, sc-6246), and mouse anti-β-actin (1:1000, Santa Cruz Biotechnology, sc-47778) 4 °C overnight, and then conjugated with the following secondary antibodies: m-IgGκ BP-HRP (1:1000, Santa Cruz Biotechnology, sc516102). Immunoblots were developed with a mixture of enhanced chemiluminescence reagents ECL (Beyotime Biotechnology, Shanghai, China) and digitally photographed in UV trans-illuminator (Bio-Rad Laboratories, CA, USA).

### Soft agar assay

Soft agar assays with 4 × 10^4^ cells in 0.3% low-melting point agarose were performed according to previous study [30]. Colony images were analyzed and captured with the EVOS flat screen microscope (Life Technologies Corp Bothell, WA, USA).

### Hematoxylin–eosin (H&E) and Masson’s trichrome staining

Tissues or ALI 3D cultures were fixed in 4% paraformaldehyde, dehydrated in a series of ethanol dilutions, and paraffin-embedded. Paraffin blocks were cut into 4 μm sections thickness and mounted on the glass slides. The sections were deparaffinized and hydrated through xylene and graded alcohol series and rinse for 5 min in tap water. The sections were stained with hematoxylin and eosin (H&E) (Zhongshan Golden Bridge Company, Beijing, China) and Masson’s trichrome staining kit (Maixin biotech company, Fuzhou, China). Morphological observation of tissues and ALI 3D cultures was photographed under the EVOS visual imaging microscope (Life Technologies).

### DAB staining

The DAB staining was performed using a commercial DAB Detection Kit (Maixin biotech company). ALI 3D cultures or tissues were fixed in 4% paraformaldehyde, embedded in paraffin, and cut into 4 μm sections. The sections were deparaffinized and hydrated through xylene and graded alcohol series and rinsed for 5 min in tap water. Antigens were retrieved by heating samples in a microwave for 15 min in citric acid buffer. The primary antibodies (1:100, rabbit anti-EpCAM, Proteintech, Chicago, IL, USA, 21050-1-AP; 1: 100, rabbit anti-Mucin1, Abcam, Cambridge, UK, ab109185; 1:100, mouse anti-P63, Abcam, ab735; 1:100, mouse anti-ERα, Santa Cruz Biotechnologies, sc-71064; 1:100, mouse anti-PR, Santa Cruz Biotechnologies, sc-398898; 1:100, rabbit anti-Vimentin, Abcam, ab137321) were incubated, respectively, on the slides at 4 °C overnight, then detected with the reaction-amplified reagent for 20 min, and conjugated with high-sensibility enzyme conjugated lgG polymer. Reactants were visualized with the fresh-prepared DAB chromogenic solutions for 3 to 5 min. Hematoxylin somatic cell staining reagent was used to counter-stain nuclei. All the coverslips were mounted on the glass slides using anti-quenching Fluoroshield™ histology mounting medium (Sigma-Aldrich) and visualized under a fluorescence microscope (BX51TF, Olympus company, Tokyo, Japan) with magnification 40 ×.

### Quantitative real-time RT-PCR

Total RNA was extracted from the tissue samples or cells using TRIzol Reagent (Life Technologies), and reverse transcription was performed using PrimeScript™II1st Strand cDNA Synthesis Kit (Takara Bio Inc., Japan). The levels of mRNA were quantitated using Toyobo Real-time PCR Master Mix (Toyobo, Japan) and analyzed in Bio-Rad Real-time PCR System (Bio-Rad) as described previously [33]. The primers for amplifying genes are shown in Table 1.

**Table 1:** List of PCR primers used in this study

Primer Names	Sequences (5′-to-3′)	Amplicon size (bp)
ERα-mRNA-F	CCCGCCTTCTACAGGTCTAAT	76
ERα-mRNA-R	CTTTCTCGTTACTGCTGGACAG	
PR-mRNA-F	CTCCGGGACCGAACAGAGT	128
PR-mRNA-R	GCGGGGACAACAACCCTTT	
β-actin-mRNA-F	GTGACGTTGACATCCGTAAAGA	245
β-actin-mRNA-R	GCCGGACTCATCGTACTCC	
Ck18-mRNA-F	CAGCCAGCGTCTATGCAGG	123
Ck18-mRNA-R	CCTTCTCGGTCTGGATTCCAC	
E-cadherin-mRNA-F	CAGTTCCGAGGTCTACACCTT	131
E-cadherin-mRNA-R	TGAATCGGGAGTCTTCCGAAAA	
TGFβ1-mRNA-F	CTTCAATACGTCAGACATTCGGG	142
TGFβ1-mRNA-R	GTAACGCCAGGAATTGTTGCTA	
Collagen I-mRNA-F	GCTCCTCTTAGGGGCCACT	91
Collagen I-mRNA-R	CCACGTCTCACCATTGGGG	
Vimentin-mRNA-F	CGGCTGCGAGAGAAATTGC	124
Vimentin-mRNA-R	CCACTTTCCGTTCAAGGTCAAG	
Fibronectin-mRNA-F	ATGTGGACCCCTCCTGATAGT	124
Fibronectin-mRNA-R	GCCCAGTGATTTCAGCAAAGG	
Avb3-mRNA-F	GGCGTTGTTGTTGGAGAGTC	138
Avb3-mRNA-R	CTTCAGGTTACATCGGGGTGA	
Hoxa10-mRNA-F	CCTGCCGCGAACTCCTTTT	203
Hoxa10-mRNA-R	GGCGCTTCATTACGCTTGC	

### Immunofluorescence assay

MEECs were grown to an appropriate density and fixed in 4% (w/v) paraformaldehyde for 15 min, permeabilized with 0.5% Triton-X-100 for 15 min, and blocked with 5% bovine serum albumin for one hour at room temperature. After blocking, cells were labeled with the primary antibodies (1:100, rabbit anti-EpCAM, Proteintech, 21050-1-AP; 1: 100, rabbit anti-Mucin1, Abcam, ab109185; 1:100, mouse anti-P63, Abcam, ab735; 1:100, rabbit anti-CD44, Proteintech, 15675-1-AP; 1:100, mouse anti-ERα, Santa Cruz Biotechnologies, sc-71064; 1:100, mouse anti-PR, Santa Cruz Biotechnologies, sc-398898; 1:100, rabbit anti-Vimentin, Abcam, ab137321), and the secondary antibodies (1:100, fluorescently labeled goat anti-mouse lgG-cy3, BA1031, Boster company, Wuhan, China) according to the manufacture’s protocol. DAPI (0.5 mg/ml, D3571, Thermo Fisher) was used to stain the nucleus. Then, the fluorescence was detected by Leica DM4000B fluorescence microscope.

### Matrigel three-dimensional (3D) culture

Single-cell suspensions of epithelial cells and HeLa cells were dispersed in a specifically differentiation medium (keratinocyte growth medium, Life Technologies) containing 5% pre-cooling Matrigel (BD Biosciences, USA). Morphogenesis assays (DAPI staining) were performed after 7 days as previously described [30, 34, 35].

### Estrogen response assay

MEECs were treated with 17β-estradiol at the concentrations of 1 nM, 10 nM, 100 nM for 24 h. Cell lysates were collected for western blotting assay. The protein samples were specifically probed with primary antibodies: mouse anti-ERα (1:1000, Santa Cruz Biotechnology, sc-71064), mouse anti-PR (1:1000, Santa Cruz Biotechnology, sc-398898), and mouse anti-β-actin (1:1000, Santa Cruz Biotechnology, sc-47778) 4 °C overnight and then conjugated with the following secondary antibodies: m-IgGκ BP-HRP (1:1000, Santa Cruz Biotechnology, sc516102). Immunoblots were colorated with a mixture of enhanced chemiluminescence reagents ECL A and B (Beyotime Biotechnology) at a ratio of 1:1 and digitally photographed in UV trans-illuminator (Bio-Rad Laboratories).

### Air–liquid interface (ALI) 3D culture

ALI cultures were performed as described previously and illustrated in Fig. 1A [30, 36, 37]. Single-cell suspensions (1 ~ 2 × 10^5^) of MEECs in 400μL growth medium (CELLnTEC Advanced Cell Systems AG, Switzerland) were dispersed into the Millicell PCF inserts (12 mm size, Millipore, Massachusetts, USA) which were placed into a 6-well plate. About 2 ml of growth medium was also dropped into the well (outside the inserts). The 6-well plate was cultured at 37 °C, 5% CO_2_. After 48 h, the growth medium was replaced with differentiation medium (CELLnTEC Advanced Cell Systems AG) inside and outside the inserts and incubated for 16 h to allow cells to form an intercellular adhesion structure. The fresh differentiation medium was changed every 2–3 days. The 3D cultures were differentiated approximately 14–19 days and harvested for histology experiments.

### Murine model of intrauterine injury and cells transplantation

8-week-old C57BL female mice were obtained from Wuhan University Center for Animal Experiment. Animal housing and killing was in accordance with guidelines of Laboratory Animal Requirements of Environment and Housing Facilities (Chinese Version). The Animal Ethics Committee of Wuhan University Center for Animal Experiment approved this study. A total of 24 mice were randomly divided into 3 groups (*n* = 16 uterine horns/group): sham operated/control group, non-transplanted/injury group, and cells-transplanted group. After injection of 2% sodium pentobarbital (45 mg/kg intraperitoneally), a vertical incision was made in the abdominal wall and the uterus was exposed. A small incision was made in each uterine horn, and the horns were traumatized using 27-gauge needle inserted two-thirds of the way through the lumen and rotated and withdrawn 10 times [3, 11, 38]. For cells-transplanted group, MEECs (1 × 10^6^) in 50 ul PBS were injected into uterine immediately after the uterine injury. For non-transplanted/injury group, 50 ul PBS was administered via intrauterine injection after injury. For the sham operated/control group, the uterine horns were left intact after exposure by an abdominal midline incision. The rectus fascia and skin of control group mice were sutured with 6–0 absorbable polyglycolic acid (PGA, Jinhuan CR631) in an interrupted fashion after PBS rinse of abdominal cavity. The uterine horns were collected at 14 days, 21 days, 30 days, and 45 days after uterus damage. The mice were killed by euthanasia with an overdose of sodium pentobarbital (200 mg/kg intraperitoneally) followed by cervical dislocation. Cardiac arrest and dilated pupils represented the death of mice before tissue sampling. The formalin-fixed paraffin-embedded tissues were sectioned longitudinally and stained with Masson’s trichrome staining. Photographs were taken under the EVOS visual imaging microscope (Life Technologies) with magnification 10 ×, 20 ×, and 40 ×. The percentage of fibrosis area (collagen area/total tissue area) was obtained by scanning the value of collagen area and total tissue area using Image J software. Numbers of glands in five different fields of vision were counted, and thickness of endometrium was measured in randomly chosen field of vision by Image J software.

### Functional recovery of injured murine endometrium

The function of the regenerated and repaired endometrium was assessed by investigating whether mice were capable of maintaining embryos development to advanced gestation [3, 39]. Additional batch of 24 female mice (8-week-old C57BL) were used and randomized into sham operated/control group, non-transplanted/injury group, and cells-transplanted group (*n* = 16 uterine horns/group). The intrauterine injury and cells transplantation were performed as above procedures. After three estrous cycles (14 days), three groups of female mice were individually bred with C57BL male mice at a ratio of 1:1. The day of vaginal suppository observation was considered as gestation day 0, and then female mice were separated from male mice (only once pregnancy). Female mice were killed with an intraperitoneal injection of pentobarbital sodium (200 mg/kg) followed by cervical dislocation at gestation day 21. The uterine horns were checked for the embryos.

### Statistical analysis

The experiments were performed in three independent tests of triplicates. Data were analyzed with GraphPad Prism 8.0. ANOVA and Dunn’s multiple comparisons test were used across three experimental groups’ comparison. All values were expressed as mean ± SD and considered significantly different when *p* value was < 0.05.

## Results

### Rapid and stable expansion of mouse endometrial epithelial cells (MEECs)

Primary mouse endometrial epithelial cells (MEECs) were isolated as described in “Materials and methods” section (Fig. 1A). The small cobble-stone-shaped epithelial colonies were observed after 24 h of plating. It is known that proliferation capacity of MEECs is extremely limited [11, 40]. Figure 1B (lower panel) shows that the primary culture of MEECs can hardly proliferate in regular culture condition (DMEM supplemented with 10% FBS). However, MEECs proliferated rapidly in the defined PECBM medium plus irradiated mouse fibroblast 3T3 cells (feeder cells) and became confluent within 72 h. The morphology of MEECs in co-culture CR condition is shown in Fig. 1B (upper panel). The cell numbers were counted at each passage, and a plot of accumulative population doublings (PDs) was constructed. The growth curve indicated the logarithmic growth rate of MEECs (Fig. 1C). More importantly, continued cultures of MEECs with more population doublings (51 PDs in 53 days) allow to generate enough endometrial epithelial cells for treating IUA. These results showed that stable cultures of MEECs could be established and expanded rapidly. The short tandem repeat (STR) analysis was performed commercially. MEECs have 10 STR loci (Fig. 1D). STR analysis verified uniqueness of MEECs and does not match any other cell lines registered in the database.

### MEECs maintain normal biological characteristics

**Fig. 2:**
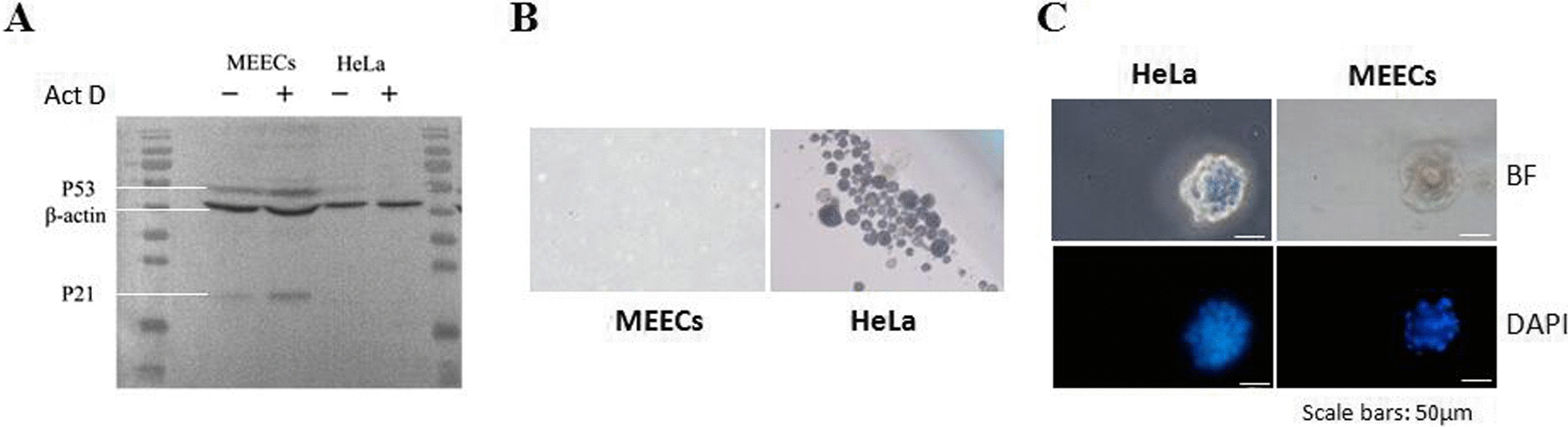
Characterization of normal biological features of MEECs. **A** Normal response to DNA damage. DNA damage was induced by treatment of cells with 0.5 nM actinomycin D (Act D) for 24 h. The response was measured by the levels of p53 protein and its downstream target molecule p21. β-actin is a loading control. HeLa cells were as the control cells. The experiment was repeated three times; the representative data were shown. **B** Non-transformed property—soft agar assay. The assay showed that MEECs did not form cell colonies in soft agar after 30 days, while cancer cells (HeLa) formed cell colonies after 10 days of culture. The morphology of colonies was observed and photographed under the microscope. Magnification 10 ×. **C** Differentiation potential in matrigel 3D culture. Single-cell suspensions of HeLa cells and MEECs were cultured in a specifically differentiation medium (keratinocyte growth medium, Life Technologies) containing 5% matrigel for 7 days as described in “Materials and methods” section. The morphology of cell aggregates was stained by 0.5 μg/ml DAPI and photographed by fluorescence microscope. Magnification 10 ×

Normal cells possess the ability to arrest cell growth when exposed to a mutagen, while tumor cells lost this normal function [37]. Next, we wanted to analyze the response of MEECs to P53-induced growth arrest induced by mimic DNA damage. MEECs were treated with actinomycin D (Act D) for 24 h, and cell lysate was collected. Figure 2A shows that both p53 and the downstream effector p21 were up-regulated in MEECs treated with Act D. However, in a cancer cell line (HeLa cells) treated with Act D, neither p53 nor p21 protein level was induced compared to untreated cells. We then evaluated the transforming property of MEECs in soft agar (anchorage-independent assay), an assay which has been widely used for testing malignant transformation of cells. MEECs did not form colonies and existed as single cells or cell debris in soft agar culture for 30 days (Fig. 2B). In contrast, anchorage-independent colonies were readily observed in 7 days with HeLa cells. The differentiation potential of normal cells is critical for their physiological functions [41]. Therefore, we took advantage of matrigel three-dimensional (3D) culture to evaluate the differentiation potential of MEECs (normal cells) and cancer cells as previous reports [24, 35, 36]. Since matrigel contains multiple factors maintaining normal homeostasis and tissue morphology [42], 3D cultures have been widely used for evaluating differentiation potential, including the group where CR technology was developed [24, 36]. “Well-organized and smooth spheres in 3D matrigel cultures” as shown in Fig. 2A of the article [24] were considered as in vitro differentiation potential; however, transformed cells or cancer cells formed non-structured and irregular-sphere aggregates [24]. As shown in Fig. 2C, we found that HeLa cells formed non-structured and irregular-sphere aggregates, since HeLa cells are tumor cells and lost the physiological function (e.g., differentiation potential). MEECs were established from normal endometrial tissue and possessed the normal response to DNA damage, and we expected that MEECs are normal cells and have normal differentiation potential. Our results demonstrated that MEECs formed well-organized and smooth surface sphere in 3D matrigel cultures (Fig. 2C). Previous studies have indicated that CR cells are adult stem-like cells and have differentiation potential in vitro assays, matrigel 3D cultures and air–liquid interface (ALI) 3D cultures, and in vivo tissue repair/regeneration [24, 25, 36], suggesting a solid foundation for treating IUA. Taken together, our results demonstrated that we were able to establish rapid and stable cultures of MEECs from mouse uterine tissue using CR technology. MEECs maintained the normal biological characteristics, normal differentiation potential, and physiological functions.

### MEECs express estrogen and progesterone receptors and respond to estrogen stimulus

**Fig. 3:**
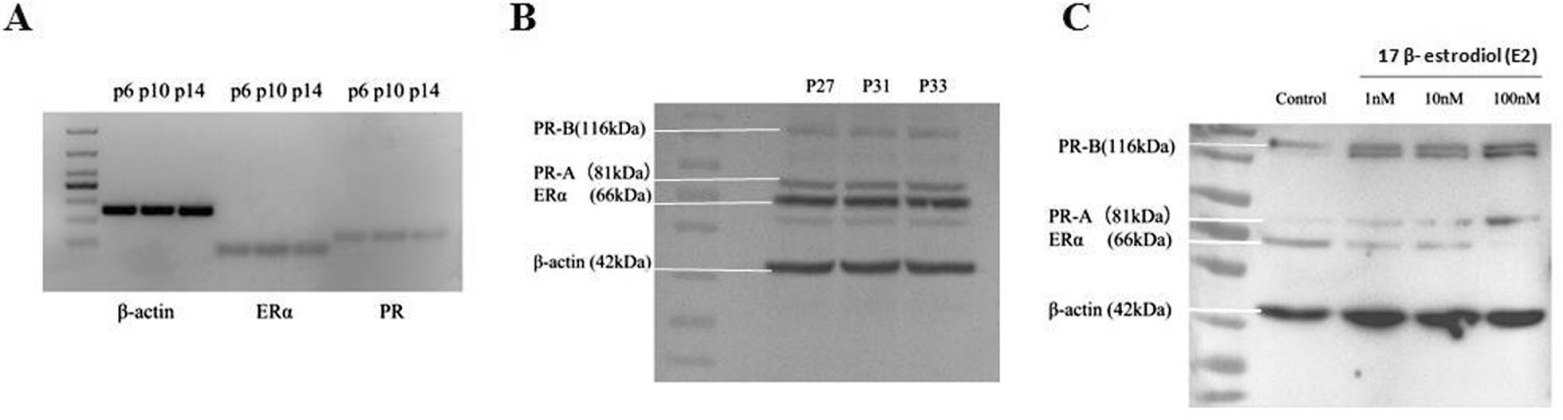
MEECs express endogenous ER and PR and respond to estrogen stimulus. **A** mRNA expression of ERα and PR in MEECs. **B** Protein expression of ERα and PR in MEECs. MEECs at different passages were harvested, and the endogenous expression of ERα and PR was analyzed by RT-PCR and western blotting. β-actin is a loading control. The experiment was repeated three times. The representative data were shown. **C** MEECs respond to exogenous estrogen stimulus. Cells were treated with 17β-estradiol at different concentrations of 1 nM, 10 nM, 100 nM for 24 h. And then cells were harvested, and the expression of ERα and PR was analyzed by western blotting. β-actin is a loading control. The experiment was repeated three times. The representative data were shown

Since the expression of ER/PR and responsiveness to estrogen (E2) and progesterone (P4) are essential for endometrial functions [43], we examined the expression of ERα and PR (PRA and PRB) in MEECs. Figure 3A and B shows that MEECs at different (even late) passages express both mRNAs and proteins of ERα and PR. It is known that PR is up-regulated by estrogen (E2) exposure, creating a fine-tuned E2/P4 feedback physiology system [44, 45]. Next, we investigated responsiveness of MEECs to exogenous E2 stimulus (Fig. 3C). MEECs were treated with 17β-estrodiol (E2) at 1 nM, 10 nM, and 100 nM for 24 h. The results showed that PR expression was up-regulated by E2 exposure compared to untreated control cells. The PR expression was increased in a dose-dependent manner. The highest induction of PR expression was observed in the cells with 100 nM E2 treatment. In contrast, the ERα expression was down-regulated after E2 treatment in a dose-dependent manner. Interestingly, the lowest level of ER expression was in the cells treated with 100 nM E2 treatment. As we know, the balance between estrogen and progesterone is critical for maintaining endometrial homeostasis. The in vitro long-term cultures of MEECs not only express endogenous ER and PR, also maintain the exquisite sensitivity to sex hormone. Thus, our results indicated that MEECs possess the most important hallmark of functional uterine endometrium.

### MEECs express tissue-specific markers and maintain lineage differentiation potential

**Fig. 4:**
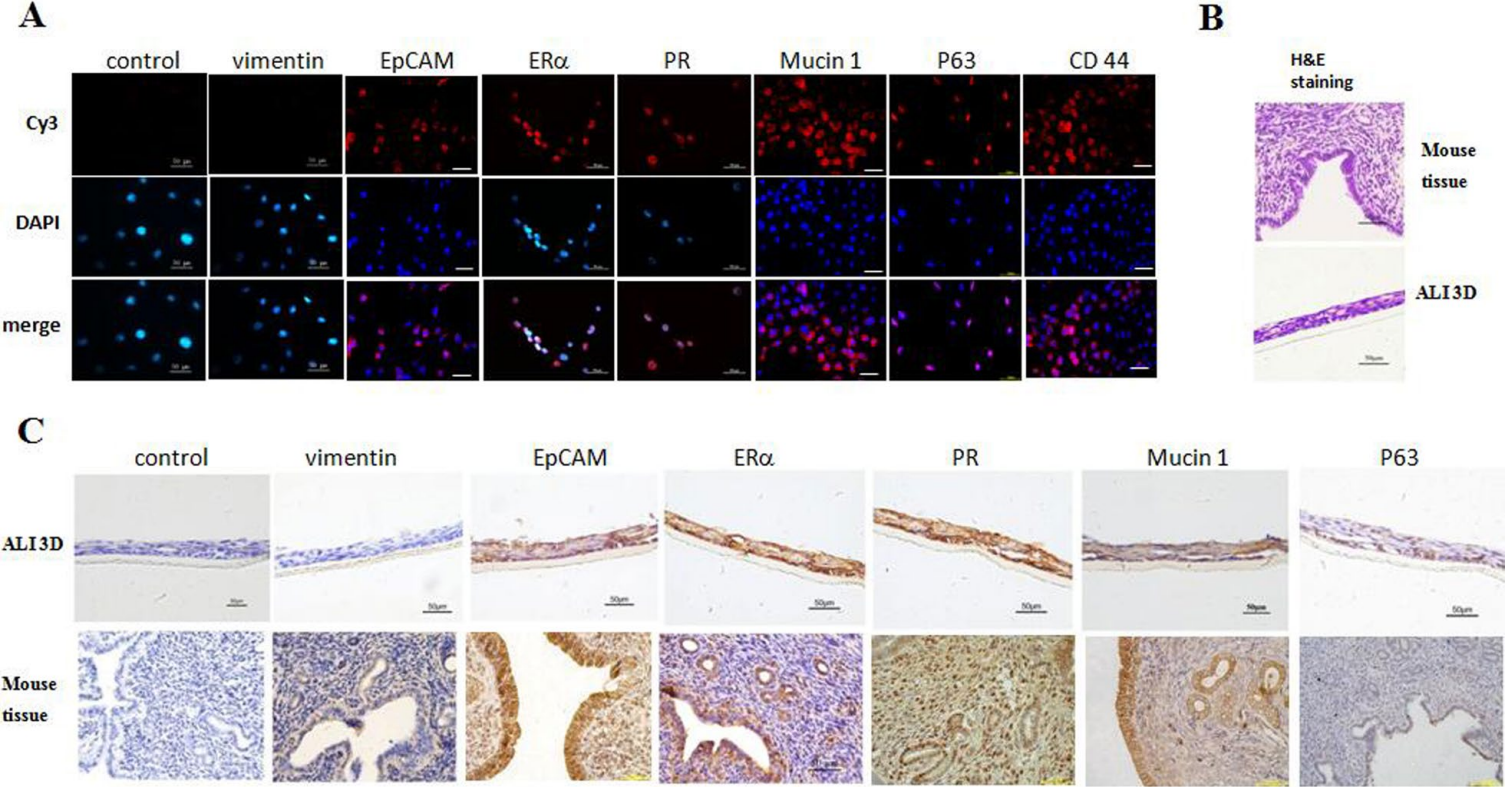
MEECs express tissue-specific markers and maintain lineage differentiation potential. **A** MEECs express tissue-specific markers. Cells were cultured on the sterile glass cover slips at an appropriate density and fixed in 4% (w/v) paraformaldehyde, permeabilized with 0.5% Triton-X-100, and labeled with the primary antibodies against Vimentin, EpCAM, ERα, PR, Mucin1, P63, and CD44, respectively. These protein markers were detected by immunofluorescence assay. The proteins were stained by second antibody goat-anti-mouse IgG—Cy3. The nuclei were stained by 0.5 μg/ml DAPI. Scale bar, 50 μm. **B** Histological structure of murine endometrium tissue and ALI 3D cultures. The cells were cultured in air–liquid interface (ALI) systems for 14 days. The murine endometrium tissue or ALI 3D cultures were fixed by 4% paraformaldehyde (w/v) and then paraffin-embedded and sectioned using standard histological procedures. The result of H&E staining was photographed under the microscope. Scale bar, 50 μm. **C** Expression of tissue-specific markers in murine endometrium tissue and ALI 3D cultures. MEECs were cultured in air–liquid interface (ALI) for 14 days. The originated endometrium tissue or ALI 3D cultures were fixed by 4% paraformaldehyde (w/v) and paraffin-embedded, sectioned, and detected by DAB staining with the specific antibodies against Vimentin, EpCAM, ERα, PR, Mucin1, and P63. Scale bar, 50 μm

In addition to the expression of ERα and PR (Fig. 4A), we also investigated the tissue origin of MEECs by detecting other uterine endometrium markers. CR method specifically expands epithelial cells and inhibits stromal fibroblasts [24]. As shown in Fig. 4A, MEECs were observed positive for luminal/glandular endometrium markers EpCAM, Mucin 1, and negative for stromal marker Vimentin [12, 23]. Our results confirmed that MEECs originated from the luminal/glandular endometrium but not the stroma. Suprynowicz et al. demonstrated that CR cells behave like adult stem cells [36]. P63 is required for the maintenance of stem cells in mouse and human epithelium, and also a specific marker for CR cells [32, 36]. CD44 is another marker of epithelial stem cells [36]. These two markers were readily detected in MEECs, although some cells were stained weak positive due to the different expression levels (Fig. 4A). It has been reported that EpCAM^+^CD44^+^ can be used to identify mouse endometrial epithelial progenitor cells [12]. Thus, these data demonstrated that MEECs were adult stem-like epithelial cells derived from luminal/glandular endometrium.

Our previous data already showed the normal differentiation potential of MEECs in 3D matrigel culture (Fig. 2C). Next, we took advantage of air–liquid interface (ALI) culture to further evaluate the morphology and structure of the endometrial epithelium differentiated from MEECs. Figure 4B shows the H&E staining of mouse uterine endometrium tissue and MEECs-derived ALI 3D culture. Both had the similar histological structure of endometrial epithelium. And the endometrium specific markers were used to verify the identical uterine epithelial specificity. Compared with mouse endometrium, MEECs-derived ALI 3D cultures expressed similar luminal/glandular endometrium markers EpCAM, Mucin 1, and ERα, PR. For the stemness marker P63, MEECs-derived ALI 3D cultures were clearly stained positive, while mouse uterine tissue was observed few positive and limited at the luminal endometrium (Fig. 4C). Taken together, our results demonstrated that MEECs maintained the tissue-specific differentiation potential which is crucial for endometrium regeneration and repair.

### Transplantation of MEECs repairs the injured endometrium morphologically

**Fig. 5:**
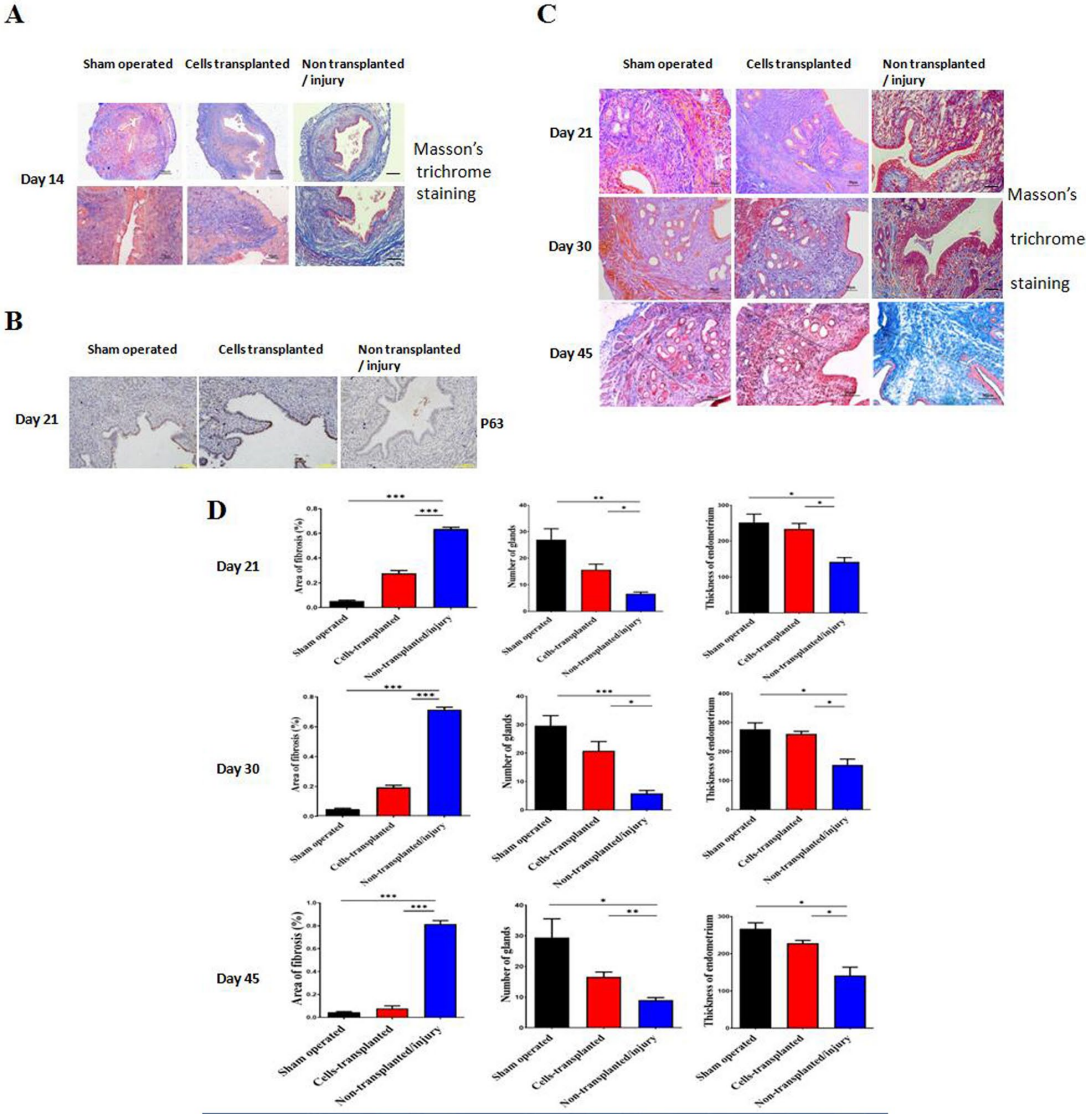
MEECs transplantation and repair intrauterine injury. **A** Trichrome-staining of murine uterine tissues. Uterine horns were collected from sham operated/control group, non-transplanted/injury group, and cells-transplanted group at three estrous cycles (day 14) after uterus damage/cell transplantation. Tissues were fixed by 4% paraformaldehyde (w/v), paraffin-embedded, and sectioned using standard histological procedures. The result of staining was photographed under the microscope. Scale bar, 50 μm. Blue staining indicated the fibrosis. **B** P63 expression in murine endometrium tissue at day 21. Sectioned tissues were detected by DAB staining. Scale bar, 100 μm. **C** Comparison of murine endometrial structure by trichrome-staining. Uterine horns were collected from sham operated/control group, non-transplanted/injury group, and cells-transplanted group at day 21, 30, and 45 after uterus damage/cell transplantation. Tissues were fixed, paraffin-embedded, and sectioned using standard histological procedures. The representative results of staining were shown. Scale bar, 50 μm. **D** Comparison of area of fibrosis, gland numbers, and thickness of endometrium. The gland numbers, thickness of endometrium, and area of fibrosis were counted or measured in relatively set views. Data were presented as mean ± SD (*n* = 3). **p < 0.01, ***p < 0.001

To investigate whether MEECs are capable of repairing injured endometrium in vivo, we constructed the experimental model of IUA mouse as described previously [3, 11, 38]. We collected the uterine tissues after three estrous cycles (day 14) and performed Masson’s trichrome staining to evaluate the endometrial structure and fibrosis. In injury group, the tightly arranged columnar endometrium structure was broke down and uterine appeared blue by Masson’s trichrome staining (Fig. 5A). In cells-transplanted group, MEECs engraftment significantly repaired the normal morphology of endometrium and reduced fibrosis lesions efficiently (Fig. 5A). DAB staining revealed that P63 positive cells in transplanted group increased significantly in the luminal epithelium and scattered in the gland epithelium, compared to few positive P63 cells in sham operated group and barely positive P63 cells in injury group (Fig. 5B). The results demonstrated the re-epithelialization of the luminal endometrium and regeneration of gland epithelium by transplantation of MEECs. Next, we investigated the different stages of tissue repair after MEECs transplantation at day 21, 30, and 45 (Fig. 5C). Quantitative data of the area of fibrosis, numbers of glands, and thickness of endometrium in three groups are shown in Fig. 5D. At day 21, transplantation of MEECs repaired luminal epithelium and inhibited fibrosis. At late stages (day 30 and 45), tightly structured luminal epithelium was observed with increased glands and thickness of endometrium, decreased fibrosis in transplanted mice, which are close to sham operated control mice (Fig. 5C and D). In contrast, the non-transplanted/injury mice exhibited much more areas of fibrosis, few numbers of glands, and less thickness of endometrium (Fig. 5D and E). Even at day 45, fibrosis lesions were not relieved in some areas of stroma (Fig. 5C). Taken together, our results demonstrated that transplantation of MEECs repaired the morphology of injured endometrium and inhibited fibrosis.

### Transplantation of MEECs restores the function of injured endometrium

**Fig. 6:**
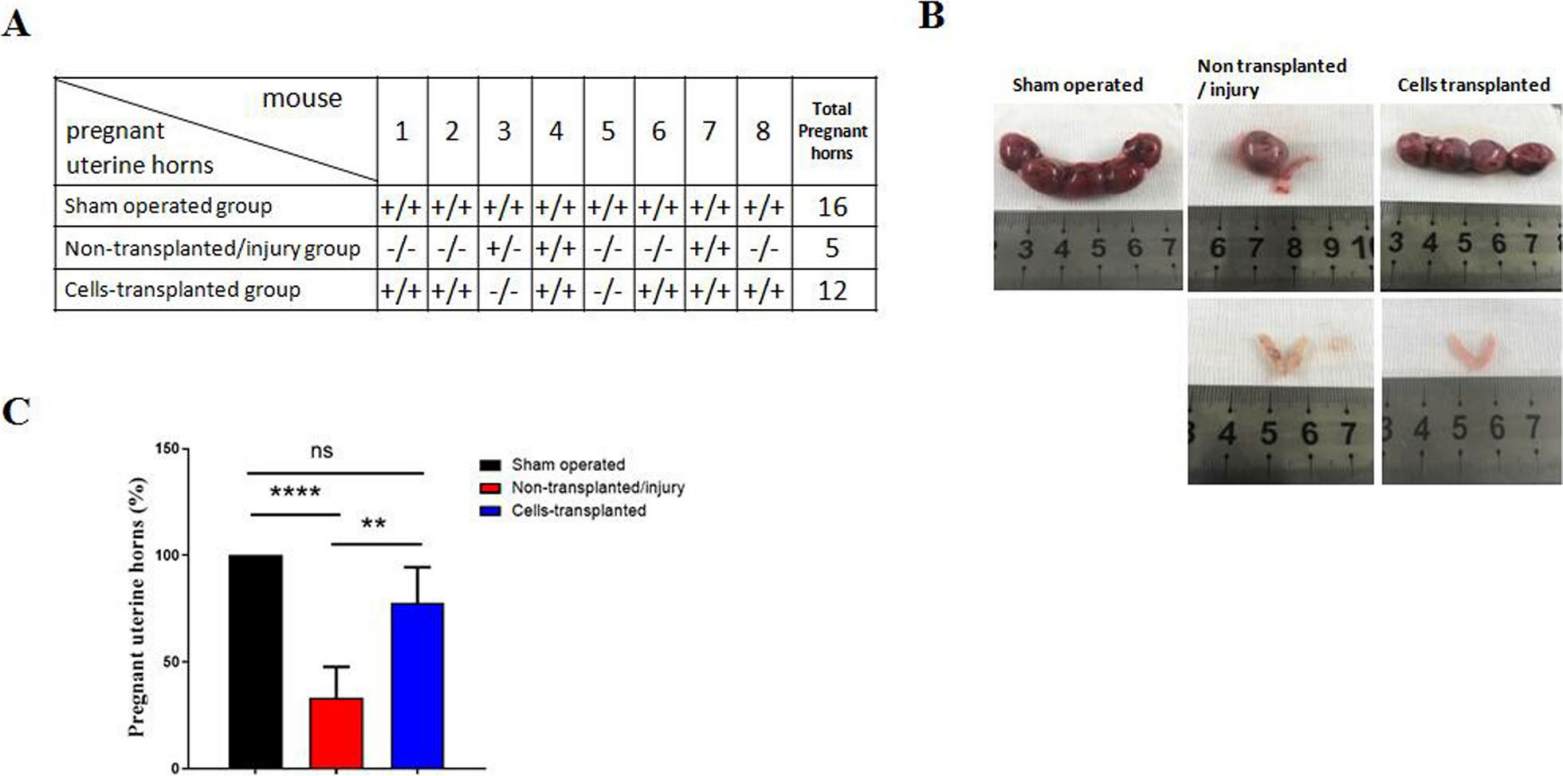
Pregnancy and embryos in mice. **A** The number of pregnant mice uterine horns. The female mice at the end of third estrous cycles after cells transplantation were bred with male mice. The day of vaginal suppository observation was considered as gestation day 0. The number of pregnant mice uterine horns were counted at gestation day 21. **B** Representative images of embryos in three different groups of mice. **C** Statistical analysis of pregnancies in different groups by Kruskal–Wallis one-way ANOVA and Dunn’s multiple comparisons test. Data were presented as mean ± SD (*n* = 3).**p < 0.01 and ****p < 0.0001

To investigate whether MEECs could restore the function of mouse injured endometrium, mice implantation was evaluated. In cells-transplanted group, 12 uterine horns were observed pregnant compared to only 5 pregnant horns in non-transplanted/injury group (Fig. 6A). It was observed symmetrical uterine horns and similar size of embryos in sham operated group. In contrast, the size of embryos varied in injury group and cells-transplanted group (Fig. 6B). Compared to non-transplanted/injury group, cells-transplanted group had a significantly improved pregnancy rate, with 75% vs 31% (*p* < 0.01), while there was no statistically significant difference between cells-transplanted group and sham operated group (Fig. 6C). These results demonstrated that transplantation of MEECs was able to restore the function of injured endometrium and dramatically improve the mice implantation.

### Key molecules regulating the repair and regeneration of endometrium by MEECs transplantation

**Fig. 7:**
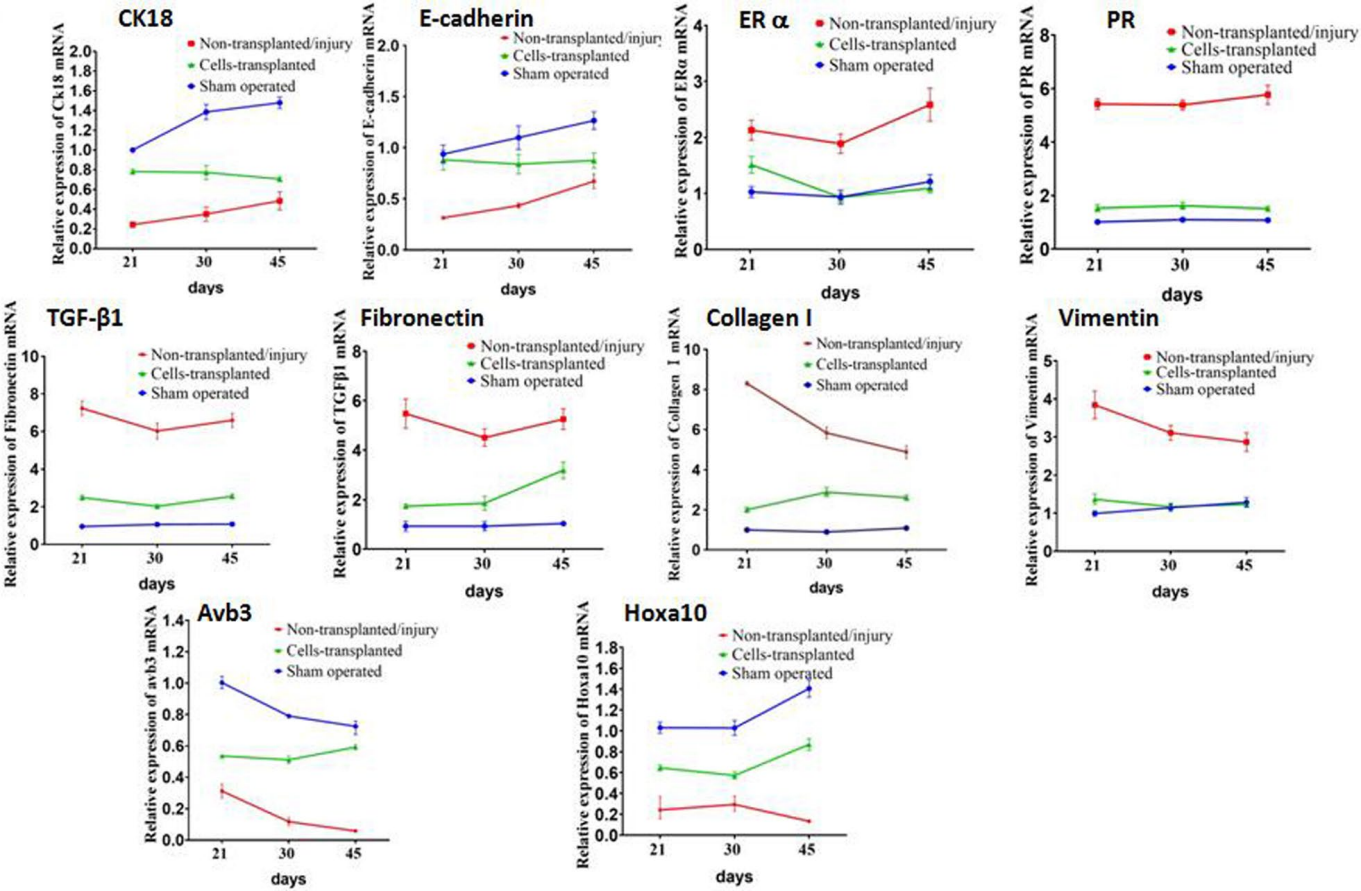
mRNA expression of key molecules during the repair of uterine endometrium by MEECs transplantation. The uterine horns of the mice in each group were collected on the 21, 30, and 45 days after the damage and transplantation. Total RNA was extracted from the uterine horns. The mRNA expression of CK18, E-cadherin, ERα, PR, TGF-β1, Collagen I, Fibronectin, Vimentin, Hoxa10, and Avb3 was detected by RT-qPCR at each time point in each group. GADPH is an internal control, and the normalized expression level is displayed in a line plot (*p < 0.05, **p < 0.01, ***p < 0.001). The statistical analysis and plotting of the data were completed using GraphPad Prism 8.0

To investigate the molecular mechanisms during the repair and regeneration of IUA mouse, total RNAs were harvested from the mice of cells-transplanted, non-transplanted/injury, and sham operated (control) groups at 21 days, 30 days, and 45 days after MEECs transplantation. RT-qPCR results showed that expression of column epithelium marker CK18 and E-cadherin was obviously down-regulated in injury group compared to that in control group (Fig. 7), while the expression of CK18 and E-cadherin in cells-transplanted group maintained relatively high and close to that in control group. The results indicate that repair of endometrium by MEECs occurred in the early phase of injury. We also found that expression of fibrosis markers (TGF-β1, Collagen I, Fibronectin, and Vimentin) was greatly up-regulated in injury group compared to that in control group (Fig. 7). MEECs transplantation significantly inhibited the fibrosis in cells-transplanted mice compared to that in injury mice (Fig. 7). These results indicated that the recovery of normal epithelial cell proliferation is essential to repair uterine endometrium.

ERα and PR were up-regulated to a high level of expression at all three time points in injured mice compared to sham operated mice (Fig. 7). The expression pattern of ERα and PR was in consistent with that of fibrosis markers. This suggested that the formation of IUA is possibly associated with abnormal up-regulation of ERα and PR.

The expression of endometrium receptivity markers (Hoxa10 and Avb3) was significantly down-regulated in injury mice and remained decreased pattern even at the very late time point (45 days) after surgery (Fig. 7). MEECs transplantation largely improved the endometrium receptivity by the consistent observation that Hoxa10 and Avb3 nearly reached to the levels in sham operated control mice. These results demonstrated that early phase repair of epithelium is critical to inhibit the fibrosis and restore the function of uterine endometrium.

## Discussion

New cases with IUA are significantly increasing numbers in recent years. This occurs in 20–25% of patients treated by dilatation and curettage after delivery [46]. In addition of curettage treatment, any uterine surgery or infection can also lead to IUA [46]. IUA is the most common cause of uterine infertility, since 25–30% of infertile women are IUA patients [47]. Current clinical procedures for moderate and severe IUA cases remain a big challenge due to the high recurrence rate after adhesiolysis [5].

Since stem cells have the capacity for self-renewal and multi-lineage differentiation, stem cell-based therapy has been proposed for treatment of IUA since 2007 [15, 48]. For example, Kilic et al. explored the mesenchymal stem cells (MSCs) to induce endometrial proliferation and angiogenesis in rat model [9]. Alawadhi et al. studied the bone marrow-derived stem cells (BMDSCs) transplantation to improve fertility in murine model [3]. Song et al. generated endometrium-like cells from human embryonic stem cells (hESCs) and repaired rat IUA [11]. Li et al. reported that they isolated human amniotic epithelial cells and treated IUA in a mouse model [7]. Since their human amniotic epithelial cells express high levels of embryonic stem cells marker SSEA4 and TRA-1-60 [49], and epithelial marker CK18 as well, full characterization of stem cells including potential of tumorigenicity should be evaluated carefully in future clinical applications.

Regeneration of endometrium in human studies has been investigated by using autologous stem cells (from bone marrow, peripheral blood, menstrual blood) [50–52]. These are several case reports or small case studies. Recently, a phase I clinical trial with 26 refractory IUA patients suggested the positive treatment of allogeneic umbilical cord-derived mesenchymal stromal cells (UC-MSCs) [53]. They indicated that the role of UC-MSCs in regeneration is to improve the multiple factors within the microenvironment but not directly involved in the reconstruction of endometrium [53, 54]. Stem cells from bone marrow, peripheral blood, menstrual blood, placenta, amniotic membrane, and other mesenchyma resources have the ability to differentiate into multiple lineages and different cell types. The exact type of stem cells that migrate and convert to endometrium has not yet been identified. The precise mechanism of how stem cells grafting improves endometrial rebuilding is still unknown [15]. For the regeneration therapy, the simple and reliable stem cell differentiation protocols need to be developed. Tumorigenicity is another major potential concern that limits the clinical utilization of pluripotent stem cells [15].

Prianishnikov proposed the “endometrial stem cells (EnSCs)” for the first time [55]. EnSCs may refer to all the sources of cells for endometrial regeneration [15, 21]. However, the precise definition and subtype of EnSCs are highly contradictory with other studies [21, 56–59]. Recently, EpCAM, E-cadherin, Mucin1, and CD44 have been identified as specific markers of endometrial epithelial stem cells in human and mouse [12, 22, 23]. However, culture and expansion of primary endometrial cells have been very difficult over decades [11, 60]. In addition to the rapid senescence of cultured endometrial cells, these cells quickly lose their phenotype and hormone responsiveness [40, 60]. In vivo, the endometrium expresses ER/PR and maintains the exquisite balance in response to E2 and P4, which is essential for endometrial functions. It has been reported that the isolated endometrial epithelial progenitor cells are sensitive to hormonal signals but do not express estrogen receptor (ER) or progesterone receptor (PR) [12]. Most recently, Yokomizo et al. reported that the very early passages (< p5) of cultured primary human endometrial epithelial cells preserved the expression of ERα and PR [61]. To obtain long-term expansion capacity of primary endometrium, organoid culture system is developed [23, 40]. Organoids are self-forming 3D reconstructions of an organ’s epithelium by using semisolid matrigel [40]. Different brands or batches of matrigel may have variable components and directly affect the success rate and characteristics of cultured endometrial organoids. The luminal cavity of endometrium is embedded inside of the organoid sphere. All these aspects largely affect functional and structural characterization of cultured endometrial organoids.

In this study, we established stable and long-term cultures of MEECs using CR technique. MEECs proliferated rapidly in CR co-culture system. MEECs retained a clear genetic background, normal biological characteristics and endometrium tissue-specific features. Most importantly, MEECs expressed estrogen and progesterone receptors and maintained hormone responsiveness even at late passages in vitro. These results demonstrated that long-term cultured MEECs possess the most important hallmark of functional endometrium which is biological basis for MEECs transplantation. It is known that CR cells retain lineage commitment and can differentiate automatically into the epithelium structure from which they derive [36, 37]. MEECs-derived ALI 3D cultures showed that MEECs were able to differentiate into the endometrium which is crucial for endometrium regeneration and repair.

Compared with chemical injury model (e.g., injection of trichloroacetic acid) [9], mechanical injury model is closer to the histological and molecular features in IUA patients [38]. Instead of scraping endometrium in rat IUA model, 27-gauge needle is more suitable to set up mouse IUA model [3, 7, 38]. Using this IUA mouse model, we observed that transplantation of MEECs significantly restores the normal morphology and inhibits the fibrosis of the endometrium. The results demonstrated that rapid epithelial repair by MEECs efficiently prevented recurrent adhesion after adhesiolysis. Recovery of endometrium by MEECs will reduce and relieve complications in IUA patients. Moreover, transplantation of MEECs dramatically improved the mice fertility and implantation. Since CR method allows to expand small sizes of samples to 1 million cells within 7 days [25], CR cells easily meet the requirement of efficacy demanded by clinical transplantation. Our results indicated that endometrial epithelial cells established by CR method may offer a novel promising strategy for clinical cell-based therapy of refractory IUA. Further basic and translational research with CR-human endometrial epithelial cells is undergoing. We expect the clinical trials with CR-endometrial epithelial cells in the near future.

## Conclusion

The present study demonstrates that endometrial epithelial cells can be established and expanded by CR approach. CR-MEECs retain a clear genetic background, normal biological characteristics, and non-tumorigenicity. CR-MEECs expressed estrogen and progesterone receptors and maintained hormone responsiveness in vitro. CR-MEECs also retain lineage commitment and possess the ability to “automatically” differentiate into the endometrium tissue in their natural environment. Transplantation of MEECs successfully repaired the injured uterine endometrium and significantly improved the pregnancy rate of IUA mice. CR endometrial epithelial cells may offer a new promising strategy for cell-based therapy in IUA clinics in a personalized or generalized manner.

## Data Availability

Not applicable.

## Abbreviations

IUA: Intrauterine adhesion
MEECs: Mouse endometrial epithelial cells
CR: Conditional reprogramming
ER: Estrogen receptor
PR: Progesterone receptor
PECBM: Primary epithelial culture basic medium
ALI: Air–liquid interface
STR: Short tandem repeat
H&E: Hematoxylin and eosin
PDs: Population doublings
Act D: Actinomycin D
3D: Three-dimensional
E2: Estrogen
P4: Progesterone
MSCs: Mesenchymal stem cells
BMDSCs: Bone marrow-derived stem cells
hESCs: Human embryonic stem cells
UC-MSCs: Umbilical cord-derived mesenchymal stromal cells
EnSCs: Endometrial stem cells
